# Errata

**Published:** 1888-10

**Authors:** 


ERRATA.—In Dr. Wooten's article for "cocti," read "cocci." In Dr. Cunningham's, read 1887 for 1877. In list of members P. M. B. A., for "Hors" read "Dr. Hons," (page 169); on page 166, in East Line Medical Society, for "editorial papers" read "elaborate papers."

